# Dynamic Adaptation of Hematological Parameters, Albumin, and Non-Esterified Fatty Acids in Saddlebred and Standardbred Horses During Exercise

**DOI:** 10.3390/ani15030300

**Published:** 2025-01-21

**Authors:** Giuseppe Piccione, Francesca Arfuso, Elisabetta Giudice, Francesca Aragona, Pietro Pugliatti, Maria Francesca Panzera, Alessandro Zumbo, Vincenzo Monteverde, Vincenzo Bartolo, Adalberto Barbera, Claudia Giannetto

**Affiliations:** 1 Department of Veterinary Sciences, University of Messina, 98121 Messina, Italy; gpiccione@unime.it (G.P.); farfuso@unime.it (F.A.); egiudice@unime.it (E.G.); fraragona@unime.it (F.A.); alessandro.zumbo@unime.it (A.Z.); 2 Clinical and Experimental Department of Medicine and Pharmacology, University of Messina, 98125 Messina, Italy; pietro.pugliatti@polime.it; 3 Department of Biomedical, Dental, Morphological and Functional Images, Specializing in Sports and Exercise Medicine, University of Messina, 98125 Messina, Italy; mfrancescapanzera@icloud.com; 4 Zooprophylactic Institute of Sicily “A. Mirri”, 90129 Palermo, Italy; vincenzo.monteverde@izssicilia.it; 5 Department of Human Pathology, University Hospital of Messina, 98125 Messina, Italy; vincenzo.bartolo@unime.it (V.B.); adalberto.barbera@unime.it (A.B.)

**Keywords:** hemogram, Jumpers, physical exercise, standardbred, platelet aggregation, albumin, non-esterified fatty acids

## Abstract

The response to exercise following a rest period may vary among horse breeds based on the importance of the hematological and hematochemical profiles of athletic horses. Ten Standardbred and ten Italian Saddlebred mares were subjected to a 4-week training program after a seasonal rest, and hematological parameters (red blood cells—RBCs; hemoglobin—Hb; hematocrit—Hct; platelets—PLTs; platelet aggregation—AG; aggregation slope—Slope; fibrinogen—Fb), as well as Albumin (Alb) and non-esterified fatty acids (NEFAs), were analyzed. Blood samples were obtained each week during the training program following a simulated exercise performed at T0 and T4 (T0pre-T0post; T1, T2, T3, and T4pre-T4post). A two-way ANOVA revealed an increase in all assessed parameters post vs. pre at T0 and T4 (*p* < 0.01) and a decrease in PLTs (*p* < 0.01) at T0 and T4 in both breeds. A significant effect of breed was observed, with higher values for RBCs and Hb at each time point (*p* < 0.001); Hct at T0 post, T2, T3, and T4 post; and NEFAs (*p* < 0.001) at T0 post and T4 post in Standardbred compared to Saddlebred horses. Positive correlations were identified among RBCs, Hb, Hct, PLTs, Alb, and NEFAs in both breeds and between AG and Slope in Saddlebred horses. Negative correlations were identified among AG and RBCs, Hb, Hct, PLTs, Alb, and NEFAs in Standardbred horses and for AG and Slope with RBCs, Hb, Hct, and PLTs in Saddlebred horses. A comparable reaction to training was observed in both breeds following the seasonal rest.

## Simple Summary

The present study evaluated the physiological response to exercise in horses after a rest period and in two different breeds. A specific 4-week training program was evaluated for 10 Standardbred and 10 Saddlebred mares. During this period, hematological parameters (red blood cells—RBCs; hemoglobin—Hb; hematocrit—Hct; platelets—PLTs; platelet aggregation—AG; aggregation slope—Slope; fibrinogen—Fb), as well as albumin (Alb) and non-esterified fatty acids (NEFAs), were evaluated. Blood samples were taken at T0pre-T0post, as well as T1, T2, T3, and T4pre-T4post. Statistical analysis revealed an increase in all parameters assessed post vs. pre at T0 and T4 and a decrease in PLTs at T0 and T4 in both breeds. Standardbred horses showed higher values for RBCs and Hb at each time point; Hct at T0 post, T2, T3, and T4 post; and NEFA at T0 post and T4 post compared to Saddlebred horses. Positive correlations were identified between RBCs, Hb, Hct, PLTs, Alb, and NEFA in Saddlebred horses and between AG and Slope in Saddlebred horses. Negative correlations were identified between AG and RBCs, Hb, Hct, PLTs, Alb, and NEFA in Standardbred horses and for AG and Slope with RBCs, Hct, and PLTs in Saddlebred horses.

## 1. Introduction

The horse should be regarded as the premier all-around athlete in the animal kingdom. Various breeds participate in competitions with different levels of exercise intensity and metabolic engagement [1,2,3,4]. The metabolic requirements of athletic horses are contingent upon their discipline and intensity, from the aerobic and anaerobic metabolism utilized in jumping events to the submaximal anaerobic exertion necessary for racehorses [5,6,7,8,9,10,11,12]. Continuous exercise elicits several alterations in the body at the cellular, tissue, and organ levels. The principal method for assessing the effectiveness of training is to analyze alterations in blood parameters during competition. It is essential to understand the hematochemical changes caused by several exercise modalities, as they indicate changes in the functions of various systems and the types of energy utilized [13].

Blood components are crucial for maintaining a high metabolic rate during exercise, supplying oxygen, water, electrolytes, minerals, and hormones to active muscles [14,15,16,17,18,19]. Analyses of the horses’ hemogram, although commonly employed in clinical practice, are frequently complex due to several endogenous and exogenous factors that markedly affect hematological parameters [20,21,22,23,24,25,26]. Among the primary endogenous determinants, the variations associated with the breed, age, and temperament of the animal are significant [27]. Among the exogenous components, sampling is crucial, as nutrition, microclimatic conditions, infectious diseases, and the training the animal undergoes, as well as the discipline with which the hemogram should be correlated, are very important [28,29]. When evaluating exercise response in humans, the necessity of understanding the physiological effects of training based on an individual’s traits has been emphasized [30]. Hematological and hematochemical characteristics were evaluated across different equestrian disciplines and breeds, demonstrating the significant diversity in the measured markers. Moreover, changes in lipid profiles in Standardbreds and Thoroughbreds were advantageous for evaluating the metabolic consumption of various substrates and the progress of the training program [31,32,33,34,35]. Previous authors have evaluated the influence of training activities on particular hematochemical parameters in racehorses [36,37] and the changes in blood parameters caused by different training intensities [38].

The physiological consequences of exercise of different durations and intensities, including evaluations of RBCs, Hb, Hct, and PLTs, were previously investigated. Hemoglobin levels were shown to increase during exercise, primarily due to changes in the hemoconcentration caused by alterations in intracellular fluid and losses during respiration and sweat. Exercise increases the number of circulating platelets. The examination of coagulation has been regarded as an effective tool for evaluating fitness in horses [39,40,41,42,43,44].

Numerous hemostatic abnormalities involving platelets, coagulation, and fibrinolysis have been documented in horses after physical activity [45,46,47,48,49,50]. The activation of the hemostatic process during physical exercise serves a protective function for the organism, protecting against microtraumas caused by the activity, and acts as an adaptive mechanism during physical training. The pivotal process in the creation of the hemostatic plug is platelet cohesiveness, generally referred to as platelet aggregation- AG [51]. Platelet aggregation serves as an indirect measure of platelet function, with platelet responses to exercise influenced by various parameters, including exercise intensity, duration, and training conditions [52,53,54,55]. Exercise substantially impacts Alb and NEFAs and contributes to a reduction in fibrinogen. Non-esterified fatty acids serve as a crucial energy source for physical performance during prolonged, low-intensity aerobic exercise [43]. Multiple studies in humans and horses have shown that Alb and NEFAs have opposite influences on platelet activation, leading to platelet aggregation, whereas their connection with Alb inhibits platelet activation. Albumin is the primary protein in mammalian serum and is essential for regulating and sustaining oncotic or osmotic pressure. This function is crucial during exercise when substantial fluid shifts occur from plasma due to changes in flow and hydrostatic pressure [56]. Additionally, Alb serves as a carrier for hydrophobic substances, including lipid-soluble hormones, bile salts, free fatty acids, calcium, ions, and certain drugs [57]. Hematological and hematochemical markers, such as AG, Alb, and NEFA levels, are acknowledged to vary in response to exercise and training [34]. A training program must maintain an appropriate equilibrium between workloads and sufficient rest intervals; its efficacy can be evaluated by the consistent assessment of specific psychological, hematochemical, and physiological indicators [58].

The present study aimed to evaluate the impact of moderate exercise, according to a 4-week training program conducted after a summer seasonal rest (one month), on RBCs, Hb, Hct, PLTs, AG, Slope, Fb, Alb, and NEFAs in two distinct horse breeds (Standardbred and Italian Jumping Saddlebred). The objective was to determine the minimum duration required to restore homeostasis post-exercise, positing that the response to exercise varies between breeds following a seasonal rest period.

## 2. Materials and Methods

### 2.1. Animals

The present study was conducted by enrolling ten 4–5-year-old Standardbred mares with a mean body weight of 450 ± 30 kg and ten 7–8-year-old Italian Saddlebred jumping mares with a mean body weight of 500 ± 25 kg, returning to activity after the summer seasonal competitive rest (one month) spent in paddocks.

Each horse was included in this study following the owner’s informed consent. Before being enrolled in this study, horses were subjected to a clinical exam including hematological and hematochemical tests to ensure their healthy status. Each horse was regularly trained before the summer rest period. None of the horses had a history of change in hemostatic parameters, and none had received any medication treatment during the preceding months. All animals were housed in individual boxes (3.50 m × 3.50 m) and were fed traditional rations based on hay (first cut meadow hay, sun-cured, late cut, average 8 kg/horse/day, 6.9% crude protein on average) and a mixture of cereals (oats and barley, 50% each, about 3.5 kg/horse/day) provided three times a day (06:00, 12:00, and 18:00). The composition of the cereal mixture was (dry matter basis) 13.0% crude protein, 20.7% crude fiber, and 3.4% ether extracts, with a calculated net energy content of 0.80 UFC (Unitè Fouragire Cheval); water was available ad libitum. Ambient temperature and relative humidity for each experimental day were continuously recorded with a data logger (Gemini Chichester, West Sussex, UK).

This research complied with the guidelines of Good Clinical Practices (EMEA, 2000) and the Italian and European regulations on animal welfare (Directive 2010/63/EU). According to national legislation, no ethics committee approval was needed for this study. All treatments, housing, and animal care reported were carried out following the standards recommended by the European Directive 2010/63/152 EU for animal experiments.

All horses involved in this study were subjected to a simulated race, different for the horses’ attitude, followed by a 4-week training program performed on a sand track that concluded with another simulated race. The characteristics of the simulated race and training program for each group are reported in Table 1 and Table 2, respectively. The same driver and the same rider performed the training program, for both the Standardbred and Saddlebred jumping horses.

### 2.2. Blood Sample Collection and Processing

**Figure 1:**
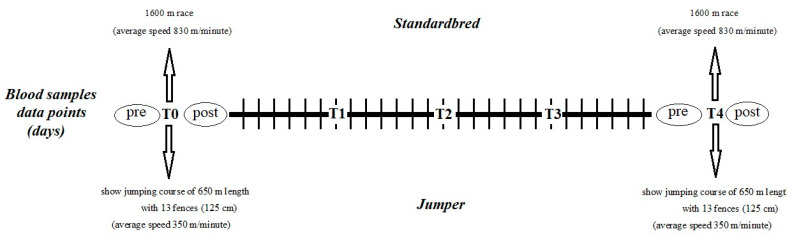
Schematic representation of the 30-day experimental protocol. The race consisted of a 1600 m run, and show jumping consisted of a 650 m course with 13 fences (six verticals 120–130 cm height; three oxers 125 cm height and 1 m deep; one triple combination of two verticals 125 cm height and 1 oxer 120 cm height).

All horses had a designated 4-week training regimen following a seasonal rest period, with two simulated races conducted at the commencement and conclusion of the 4-week training program, as in previous studies [59]. Blood samples were obtained via jugular venipuncture using tubes (Terumo Corporation, Tokyo, Japan) containing ethylenediaminetetraacetic acid (EDTA), tubes with 3.8% sodium citrate (1 part citrate/9 parts blood), and tubes with a clot activator, both before and after the initial race (T0pre-T0post), weekly for three data points (T1-T2-T3), and before and after the final race (T4pre-T4post), conducted by the same veterinarian at a consistent time of day (Figure 1).

Upon collection, all samples were promptly sent to the laboratory, and blood samples treated with EDTA were utilized to conduct a blood smear [41]. A manual platelet count was conducted to confirm and authenticate the computerized count. EDTA tubes were maintained at 4 °C and tested within 2 h after collection using an automated hematology analyzer (HeCo Vet C, SEAC, Florence, Italy) to evaluate RBCs, Hb, Hct, and PLTs.

Platelet aggregation was assessed using blood samples collected in vacuum tubes (Terumo Corporation, Tokyo, Japan) containing 3.8% sodium citrate. Platelet-rich and platelet-poor plasma were obtained through centrifugation. Platelet-rich plasma (PRP) was prepared by centrifuging samples at 300× *g* for 20 min within 15 min of collection. The resulting PRP was extracted with a plastic transfer pipette and transferred into plastic containers. To create platelet-poor plasma (PPP), the initial blood sample tubes were re-centrifuged at 4500× *g* for 15 min, and the resulting PPP was extracted and put into plastic containers. Platelet aggregation was subsequently evaluated using Anti-poly-ADP-ribose binding reagent (Mascia Brunelli S.p.a., Milano, Italy) at a dose of 1 μM as an agonist to induce platelet activation. Fifteen microliters of ADP was combined with 290 microliters of PRP while stirring, utilizing a dual-channel aggregometer (Clot 2, SEAC-Radim, Company, Florence, Italy). The results were presented as percentage aggregate. The same operator confirmed that the platelet count in the acquired PRP exhibited an increase ranging from 2.8 to 4.5 times that of whole blood, as indicated by prior investigations conducted on horse species [58,59,60,61]. The AG response was assessed using two parameters: the highest aggregation level and the initial aggregation velocity (Slope). The highest degree of aggregation was ascertained by measuring the peak height of the aggregation wave during a four-minute interval, commencing at the initiation of platelet aggregation. The maximum degree of aggregation was quantified as a percentage (%) of the highest potential variation in light transmission. Slope was ascertained by drawing a tangent line through the steepest linear segment of the aggregation trace and calculating the slope from a certain point along the curve. The gradient of this tangent was articulated in percentage per minute (%/min).

Blood samples for plasma fibrinogen concentration assays were collected in sodium citrate and promptly centrifuged at 3000× *g* for 15 min at ambient temperature. Fibrinogen levels were measured in the collected plasma using a specialized kit designed for the SEAC Clot 2 coagulometer (SEAC, Florence, Italy). The tubes with clot activator were allowed to stand at room temperature for 20 min and subsequently centrifuged at 3000 rpm for 10 min.

The resulting sera, which were neither lipemic nor hemolyzed, were analyzed for Alb and NEFA concentration utilizing commercially available kits (albumin, Byosistems, Reagents and Instruments, Barcelona, Spain; NEFAs, Randox, Crumlin, UK) with an automated ultraviolet–visible spectrophotometer (model Slim SEAC, Florence, Italy). All calibrators and samples were analyzed in duplicate. Samples demonstrated parallel displacement relative to the standard curve. The intraassay coefficient of variation was less than 5%.

### 2.3. Statistical Analysis

Data were expressed as mean ± standard deviation. Data were normally distributed (*p* > 0.05, Shapiro–Wilk test). Two-way analysis of variance (ANOVA) for repeated measures was applied to the investigated parameters to identify statistical differences due to experimental conditions (time points) and horses’ groups.

The Pearson correlation coefficient (r) was analyzed in order to consider the multiple correlation of the variation of each analyzed parameter based on the exercise protocol response, using the statistical software GraphPad Prism 9v (GraphPad Software, San Diego, CA, USA). Values of *p* < 0.05 were found to be statistically significant.

## 3. Results

**Figure 2:**
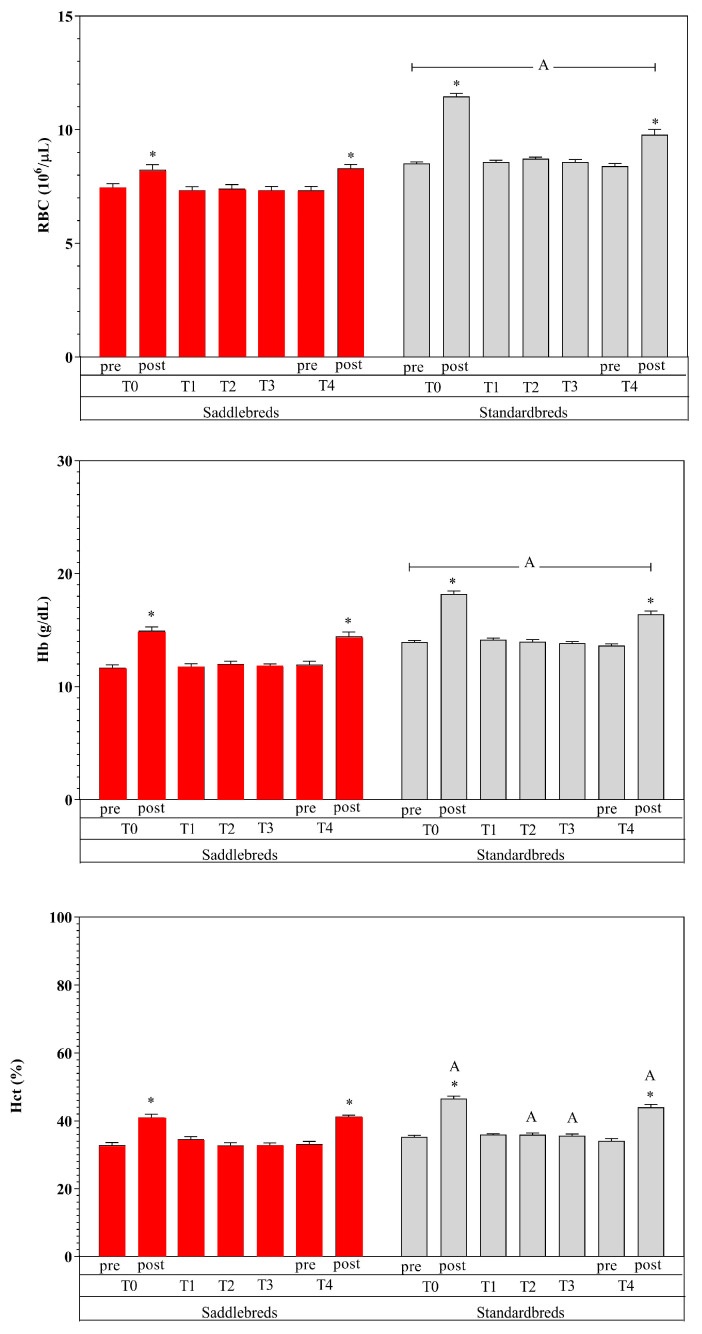
Mean values ± standard deviations of RBCs, Hb, and Hct, shown in their standard units, together with the statistical significance (*p* < 0.05) determined through post hoc comparison. * denotes the statistical impact of exercise (post versus pre) in Jumpers (red) and Standardbred (grey). The letter A delineates the significant differences between the two breeds.

**Figure 3:**
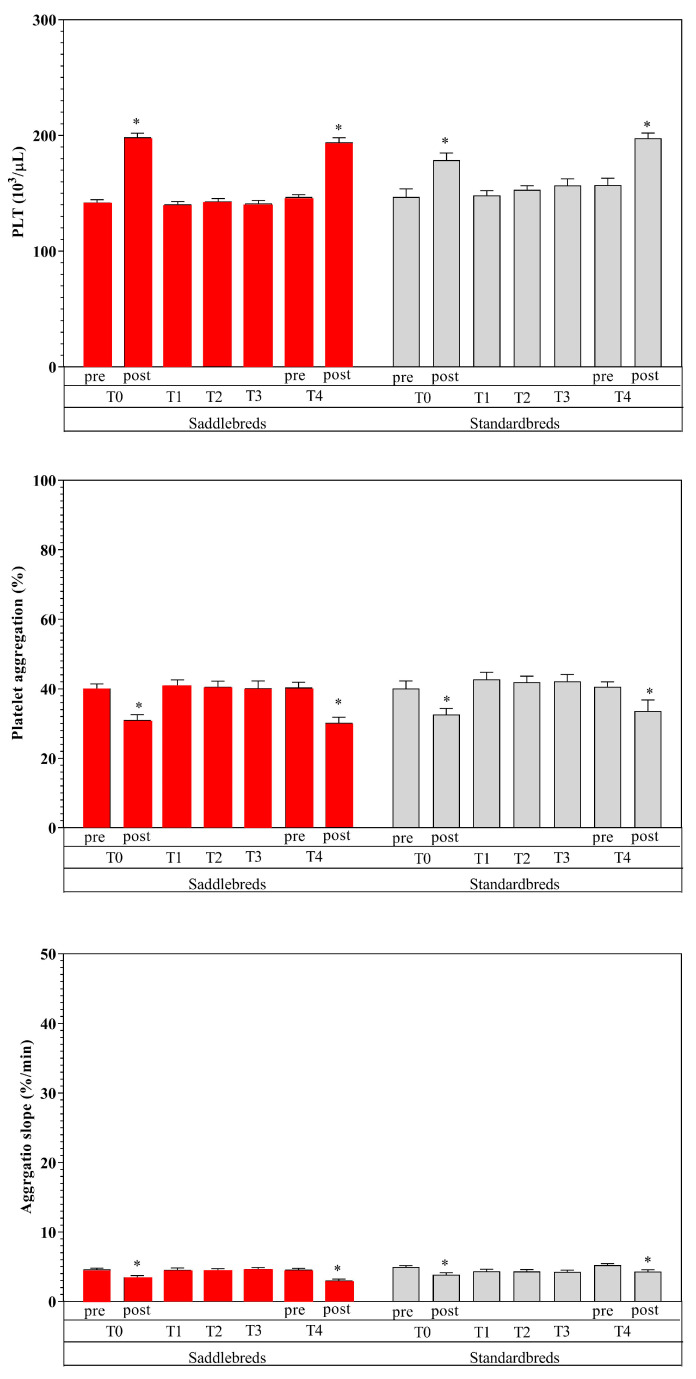
Mean values ± standard deviations of PLTs, Slope, and AG, shown in their standard units, along with the statistical significance (*p* < 0.05) determined through post hoc comparison. * denotes the statistical effect of exercise (post versus pre) in Jumpers (red) and Standardbred (grey).

**Figure 4:**
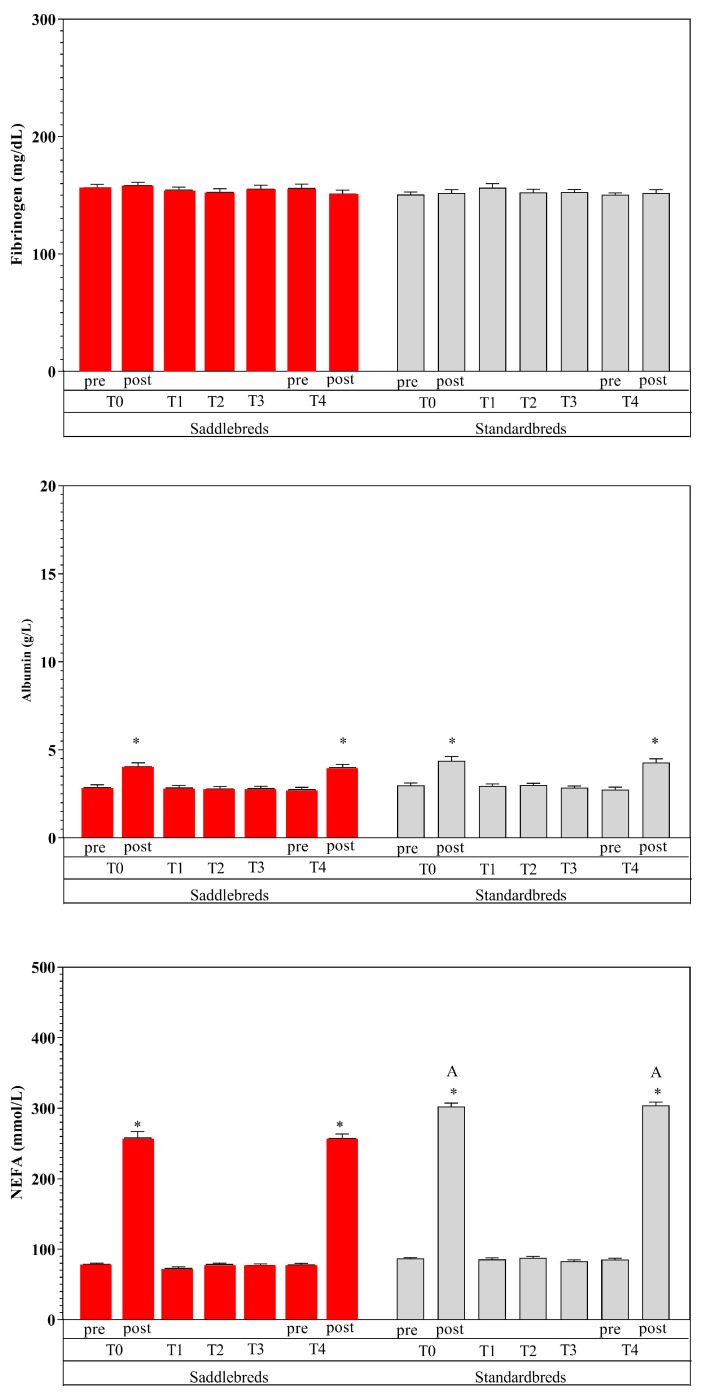
Mean values ± standard deviations of Fb, Alb, and NEFAs expressed in their standard unit, together with the statistical significance (*p* < 0.05) through post hoc comparison. * denotes the statistical effect of exercise (post versus pre) in Jumpers (red) and Standardbred (grey). The letter A delineates the significant differences between the two breeds.

Table 3 shows the mean values of the examined parameters, given in their standard units, together with the statistical significance determined through post hoc comparison (Figure 2, Figure 3 and Figure 4). The utilization of two-way ANOVA for repeated measures indicated a statistically significant effect of exercise and breed.

Specifically, an elevation in circulation levels of all examined markers was noted due to exercise (post-exercise vs. pre-exercise) (*p* < 0.01) at T0 and T4, with the exception of PLTs and Fb. Platelets exhibited a statistically significant reduction post-exercise (*p* < 0.01) at T0 and T4, but Fb displayed no alterations. Significantly increased values were seen in Standardbred horses compared to Saddlebred jumping horses for RBCs and Hb at each time point (*p* < 0.0001) and for Hct at T0 post, T2, T3, and T4 post (*p* < 0.0001). Higher NEFA post-exercise values were recorded in Standardbred compared to Saddlebred jumping horses (*p* < 0.001).

**Figure 5:**
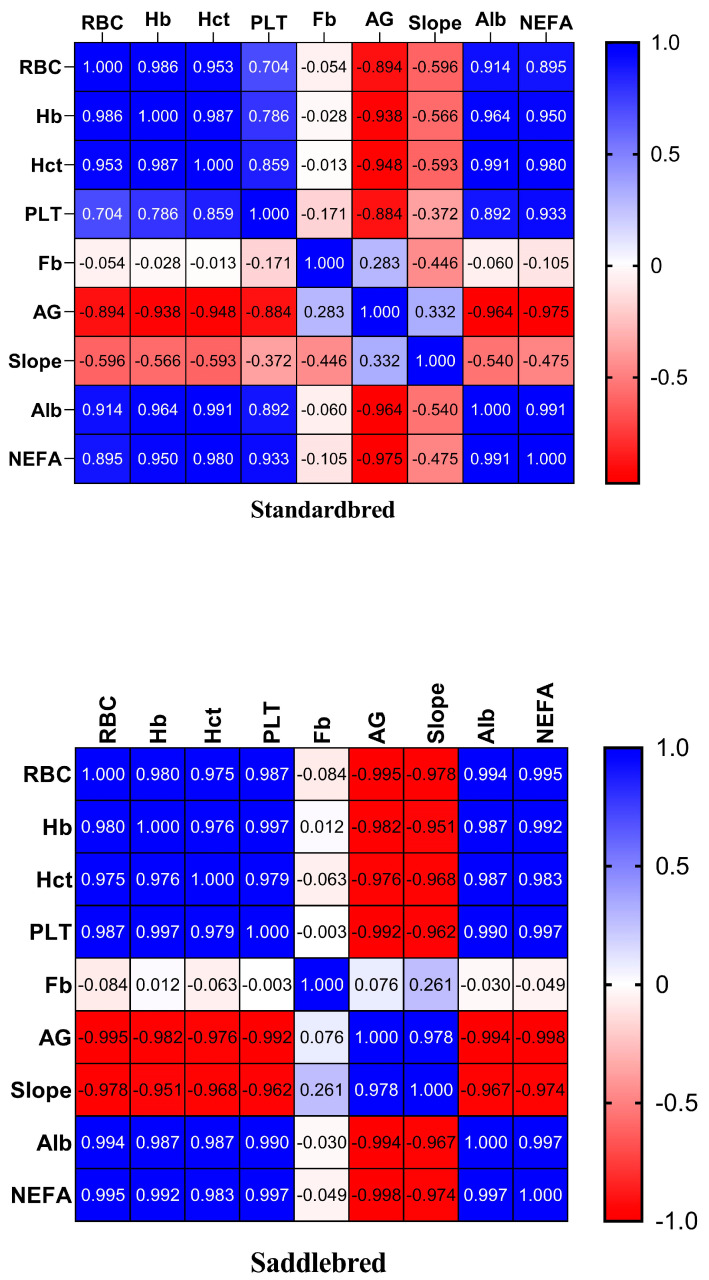
Heat map of correlation analysis (r-values) of hematological parameters (RBCs, Hb, Hct, PLTs, AG, Slope, and Fb), Alb, and NEFAs observed in Standardbred and Saddlebred horses. Each heatmap reports the r coefficient of each Pearson correlation matrix from −1 to +1.

The heat map of correlation analysis showed a significant multiple relationship between all the investigated parameters during the experimental period in both breeds. Positive multiple correlations were identified among RBCs, Hb, Hct, PLTs, Alb, and NEFAs in both breeds. AG exhibited a positive connection with Slope in Saddlebred horses. Negative correlations were identified between AG and RBCs, Hb, Hct, PLTs, Alb, and NEFAs in Standardbred horses, and AG and Slope were negatively correlated with RBCs, Hb, Hct, and PLTs in Saddlebred horses (Figure 5).

## 4. Discussion

Hematology and hematochemistry are essential instruments for evaluating the functionality of various body systems in athletic horses. The main aim of blood analyses in a trained horse is the comprehensive assessment and oversight of its general fitness. The current study revealed a substantial effect of exercise on all assessed parameters, excluding PLTs and Fb levels in both breeds. The levels of all assessed parameters were within the physiological range for horses and corresponded with the results of Piccione et al. (2008, 2014) [49,50,51,52,53,54,55,56,57,58,59].

Moderate exercise performed during the 4-week training program after a seasonal rest showed an effect on hematological and hematochemical parameters. In particular, special attention must be given to alterations in red blood indices due to the volatile nature of red blood cells [1,61]. In the post-exercise data point, a significant increase in RBCs, Hb, and Hct was observed, in both breeds [62]. This increase may be due to the secretion of catecholamines during physical exertion. The alteration in red blood cell counts during exercise results not only from the splenic release of red blood cells but also from the displacement of water from plasma during physical activity [63,64,65,66]. The elevation of RBCs, Hct, and Hb following exercise indicates the physiological adaptations necessary for muscle oxygenation, metabolite supply, and catabolite removal [65,66,67]. The elevation in Hct represents a discernible rise in blood viscosity. Fedde and Wood [68] indicated that the apparent blood viscosity in exercising horses is reduced. The elevation in Hct was correlated with an augmentation in cardiac output and the mean blood flow velocity, alongside an increase in shear rate and a simultaneous decrease in apparent blood viscosity [34,41]. The perceived viscosity of blood enhances oxygen transport capacity by increasing circulating RBCs without a corresponding rise in cardiac workload [9,69]. We can postulate that the renowned release of catecholamines during exercise not only affected the splenic release and augmented the oxygen-carrying capacity, thereby enhancing RBCs, Hct, and Hb values post-exercise, but also affected the quantity of circulating PLTs, which significantly increased after exercise in both groups. Contrary to the rise in PLTs post-exercise, a reduction in AG rate and Slope was noted following exercise, at both T0 and T4, in Standardbred and Saddlebred horses. The available data about the impact of physical exercise on clotting factors are not singular. Reports indicate hypocoagulative, hypercoagulative, and unaltered coagulative states. Certain writers ascribed these variations to the many protocols employed, including different training schedules, breeds, animal health status, and conducted analyses [39]. It has also been shown that AG may be influenced more by noradrenaline increasing than adrenaline after exercise, likely due to the downregulation of adrenergic receptors [9]. A reduction in AG and Slope post-exercise has also been ascribed to the stimulation of endothelial cells to release prostacyclin and nitric oxide by noradrenaline, both recognized as powerful inhibitors of platelet aggregation [70]. The activity of platelet adhesion is affected by the combined effects of several sticky and soluble agonist receptors. It has long been established that AG generally occurs at locations of flow disruption following vascular injury; nevertheless, it is suspected that this phenomenon is directly induced by the buildup of soluble platelet agonists at these disturbed flow sites [71]. Additional indicators of equine fitness exhibited dynamic fluctuations during the monitoring period. The present increased blood Alb levels after exercise may result from protein fluxes and a diminished intravascular fluid volume due to fluid movement from intravascular to extravascular compartments or fluid loss via perspiration. Albumin is the primary osmotically active plasma protein, due to its abundance and diminutive size, accounting for approximately 75% of plasma’s osmotic activity. It functions as a crucial storage reservoir for proteins and transporters of amino acids and hydrophobic compounds, including NEFAs which showed a similar behavior [57]. Diverse factors regulating energy homeostasis influence the mobilization and utilization of NEFAs, which are essential for optimizing physical performance during aerobic activities as free fatty acids are liberated from adipose tissue and transported via the circulation to the muscles, where they serve as fuel for muscular contraction [72]. Free fatty acids compete for binding to Alb; hence, the increase in NEFA content during exercise results from elevated adrenergic tone that stimulates subcutaneous lipolysis [35]. The increase in NEFA levels seen after exercise indicates a metabolic response of the horse to physical activity and can be ascribed to the recognized ability of horses to utilize lipids as an energy source [73,74]. It was suggested that any condition leading to increased NEFA levels may contribute to platelet hyperactivity [73]. Nevertheless, this behavior was followed by a decrease in AG and Slope after exercise in both breeds, as observed by similar previous studies [72].

A notable disparity in the levels of RBCs, Hct, Hb, and NEFAs was detected between Standardbred and Saddlebred jumping horses in response to exercise. Red blood cell indices exhibit considerable variability among horse breeds. This relates to characteristics such as the expansion of plasma volume, which is observed in endurance-trained animals but not in Thoroughbred or Quarter horses, suggesting that exercise type and intensity can considerably influence red cell indices [62]. A similar response has been noted in both Standardbred and Saddlebred jumping horses. Standardbred and Saddlebred horses typically participate in different sport disciplines (middle-distance racing and jumping); however, both possess the capacity to derive energy from aerobic and anaerobic pathways [73]. Aerobic training is known to reduce blood serum catecholamine levels, downregulate lipolysis, decrease the mobilization of NEFAs, and enhance energy use from NEFAs by muscle post-training [73].

The increasing values of hematological parameters after exercise showed a positive correlation with Alb and NEFAs, highlighting the enhancement of the oxygen capacity that is a crucial element of the horse’s elevated aerobic capacity and affecting both splenic contraction and AG in both breeds [63]. A negative correlation was observed among the hematological parameters, AG, and Slope, highlighting the diminished aggregation capacity during exercise. This confirms that elevated hematological and hematochemical parameters can inhibit AG in horses during exercise in both breeds [72].

In the Standardbred horses, the period of time required for the restoration of homeostasis after exercise performed during the experimental period seems to vary. RBCs, Hct, and Hb statistically showed a difference from the results obtained for Saddlebred horses. This could suggest a different physiological adaptation mechanism to exercise according to the two breeds, taking into account a genetic influence that should be further investigated considering previous studies in which this influence was observed, showing a significant difference in recovery during sport performances, and a genetic basis appropriate for selection [74].

In the present study, a range of different ages were analyzed in the different breeds, and certainly, this could be a limitation for the present study. However, few studies are present regarding the influence of different ages and performances on hematological and hematochemical parameters in horses. In fact, it is known that age does not influence other physical, hematological, and hematochemical parameters in endurance horses [75].

## 5. Conclusions

An effect of exercise was observed in the different hematological parameters analyzed in both breeds, and a difference in the effect of training on the hematological profile analyzed was observed among the breeds considered. Moreover, it appears that the period required for the restoration of homeostasis following moderate exercise carried out after rest and after 4-week training may vary according to the influence of the breed.

The experimental results effectively enhance our understanding of alterations in the horse’s internal environment during exercise, providing a comprehensive overview of the physiological responses in different breeds and disciplines, both after a rest period and after a targeted prolonged training regimen.

More studies would be desirable for the evaluation of the homeostatic response to exercise under different training programs, but especially the evaluation of the same training protocol subjected to higher intensities would be of interest. Based on the homeostatic response of different breeds of sport horses, this information will be useful in establishing appropriate management and training protocols, considering a seasonal rest period, ensuring health conditions and proper preparation of animals for preventive purposes so as to prevent physiological imbalances or pathological outbreaks during athletic performances based on discipline and breed.

## Figures and Tables

**Table 1:** Weekly training schedule performed by the ten Standardbreds during the protocol period. Speed = distance/time.

Day per Week	Gait	Time (min)	Speed (m/s)
2	Walk	15	5.0
	Slow trot	20	
	Walk	15	
2	Walk	15	5.0
	Slow trot	5	7.0
	Moderate trot	10	
	Walk	15	
1	Walk	15	5.0
	Slow trot	5	10.0
	Moderate/high trot	10	
	Walk	15	
1	Walk	15	11.0
	Interval training	15	
	Walk	15	
1	Rest	-	-

**Table 2:** Weekly training schedule performed by the ten jumper horses during the protocol period.

Day per Week	Gait	Time (min)	Height Obstacles (cm)
3	Walk	10	
	Trot	25	
	Canter	15	
	Walk	10	
2	Walk	10	90–100
	Trot	20	
	Canter	15	
	Walk	10	
1	Walk	15	125
	Trot	5	
	Canter	10	
	Walk	15	
1	Rest	-	-

**Table 3:** Mean values and ranges of all the significant values obtained with their standard units in Saddlebred and Standardbred horses monitored before the training program at T0 (pre–post), T1, T2, T3, and T4 (pre–post).

	Saddlebred							Standardbred						
	T0		T1	T2	T3	T4		T0		T1	T2	T3	T4	
	pre	post				pre	post	pre	post				pre	post
RBCs (10^6^/μL)	7.46 (6.84–8.37)	8.24 (7.38–9.71)	7.35 (6.5–8.19)	7.4 (6.59–8.15)	7.33 (6.7–7.77)	7.34 (6.59–8.41)	8.3 (7.5–8.87)	8.51 (8.23–8.81)	11.47 (10.8–11.9)	8.57 (8.08–9.01)	8.72 (8.2–8.9)	8.58 (8.03–9.01)	8.39 (8.07–9.35)	9.77 (8.6–10.8)
Hb (g/dL)	11.7 (10.7–13.5)	14.96 (12.7–15.5)	11.79 (10.6–12.6)	12.01 (11–13.6)	11.75 (10.8–13.6)	11.95 (11.1–13.3)	14.46 (12.2–15.9)	13.96 (13.5–14.4)	18.2 (16.9–19.3)	14.15 (13.5–14.8)	13.9 (13.1–14.9)	13.85 (13–14.6)	13.64 (13–14.4)	16.42 (15.3–17.6)
Hct (%)	32.84 (28.8–35.7)	41.06 (38.3–46)	34.57 (31.6–37.6)	32.74 (31–35.8)	32.8 (29.7–36.3)	33.22 (29.9–36.5)	41.21 (38.6–42.6)	35.35 (34–37.1)	46.59 (43.3–49.8)	36 (34.7–37.3)	35.9 (33.6–38)	35.66 (34.2–38.6)	34.14 (31.1–37.4)	44.06 (40.4–48.1)
PLTs (10^6^/μL)	142.1 (133–153)	198.6 (177–209)	140.6 (129–155)	143.2 (131–157)	141.3 (131–157)	146.8 (139–155)	194.1 (174–215)	147 (118–188)	178.6 (148–209)	148.3 (134–171)	153 (142–177)	156.8 (132–187)	157.31 (120–175)	197.8 (180–223)
AG (%)	40.1 (35–46)	31 (21–38)	41 (32–45)	40.6 (33–48)	40.2 (30–47)	40.4 (34–47)	30.2 (22–38)	40.1 (30–51)	32.6 (28–42)	42.7 (33–50)	41.9 (30–49)	42.2 (30–50)	40.6 (35–48)	33.65 (24–51)
Slope (%/min)	4.62 (4–5.5)	3.5 (2.5–5)	4.55 (3–6)	4.5 (3–5.5)	4.67 (3.25–4.75)	4.57 (3.75–5.5)	3.02 (2–4)	4.96 (4.25–6.5)	3.88 (3–4.56	4.36 (2.5–5.5)	4 (3–5.5)	4.26 (3.5–5.5)	5.24 (4.5–6)	4.28 (3–5.5)
Alb (g/L)	2.88 (2.36–3.8)	4.07 (3.1–4.8	2.87 (2.4–3.6)	2.82 (2.15–3.44)	2.84 (2.3–3.24)	2.77 (2.07–3.31)	4.03 (3.2–4.8)	3 (2.3–3.6)	4.39 (3.1–5.5)	2.95 (2.15–3.5)	3.0 (2.5–3.4)	2.86 (2.5–3.3)	2.74 (2–3.5)	4.29 (3–5.5)
NEFAs (mmol/L)	79 (74–89)	258.5 (220–301)	73.5 (63–78)	79 (72–85)	77.7 (72–85)	78.5 (72–86)	257.8 (236–278)	86.9 (79–89)	302.4 (278–324)	85.5 (75–97)	87.6 (75–99)	83.2 (75–96)	85.4 (76–94)	304.2 (283–324)

## Data Availability

The data presented in this study are available on request from the corresponding author. The data are not publicly available due to privacy reasons.

## References

[1] Hinchcliff K.W., Kaneps A.J., Geor R.J. (2004). Equine Sport Medicine and Surgery.

[2] Klein D.J., McKeever K.H., Mirek E.T., Anthony T.G. (2020). Metabolomic Response of Equine Skeletal Muscle to Acute Fatiguing Exercise and Training. Front. Physiol..

[3] Aragona F., Di Pietro S., Arfuso F., Fazio F., Piccione G., Giudice E., Giannetto C. (2022). Correlation between Ocular and Rectal Temperature with Intra Ocular Pressure in Horse during Exercise. Animals.

[4] Medica P., Giunta R.P., Bruschetta G., Ferlazzo A.M. (2020). The Influence of Training and Simulated Race on Horse Plasma Serotonin Levels. J. Equine. Vet. Sci..

[5] Coelho C.S., Sodre T.D.R.P., Sousa L.N., Siqueira R.F., Manso Filho H.C., Aragona F., Fazio F. (2021). How Much Energy Vaquejada Horses Spend in a Field Simulation Test?. Animals.

[6] Kirsch K., Fercher C., Horstmann S., von Reitzenstein C., Augustin J., Lagershausen H. (2021). Monitoring Performance in Show Jumping Horses: Validity of Non-Specific and Discipline-Specific Field Exercise Tests for a Practicable Assessment of Aerobic Performance. Front. Physiol..

[7] Fazio F., Aragona F., Piccione G., Pino C., Giannetto C. (2023). Cardiac Biomarker Responses to Acute Exercise in Show Jumping Horses. J. Equine. Vet. Sci..

[8] Sainas G., Melis S., Corona F., Loi A., Ghiani G., Milia R., Tocco F., Marongiu E., Crisafulli A. (2016). Cardio-Metabolic Responses during Horse Riding at Three Different Speeds. Eur. J. Appl. Physiol..

[9] Jones C.J.H., Defily D.V., Patterson J.L., Chilian W.M. (1993). Endothelium-dependent relaxation competes with α1- and α2 adrenergic constriction in the canine epicardial coronary microcirculation. Circulation.

[10] Kiely M., Warrington G.D., McGoldrick A., Pugh J., Cullen S. (2020). Physiological Demands of Professional Flat and Jump Horse Racing. J. Strength Cond. Res..

[11] Munsters C.C.B.M., van Jwaarden A., van Weeren R., Sloet an Oldruitenborgh-Oosterbaan M.M. (2014). Exercise testing in warmblood sport horses under field conditions. Vet. J..

[12] Gavazza A., Delgadillo A.J., Gugliucci B., Pasquini A., Lubas G. (2002). Haematological Alterations Observed in Equine Routine Complete Blood Counts. A Retrospective Investigation. Comp. Clin. Path..

[13] De Miranda R.L., Mundim A.V., Saquy A.C.S., Costa Á.S., Guimarães E.C., Gonçalves F.C., Carneiro E Silva F.O. (2009). Biochemical Serum Profile of Equines Subjected to Team Penning. Comp. Clin. Pathol..

[14] Maśko M., Domino M., Jasiński T., Witkowska-Piłaszewicz O. (2021). The Physical Activity-Dependent Hematological and Biochemical Changes in School Horses in Comparison to Blood Profiles in Endurance and Race Horses. Animals.

[15] Muñoz A., Riber C., Santisteban R., Rubio M.D., Agüera E.I., Castejón F.M. (1999). Cardiovascular and Metabolic Adaptations in Horses Competing in Cross-Country Events. J. Vet. Med. Sci..

[16] Zobba R., Ardu M., Niccolini S., Cubeddu F., Dimauro C., Bonelli P., Dedola C., Visco S., Pinna Parpaglia M.L. (2011). Physical, Hematological, and Biochemical Responses to Acute Intense Exercise in Polo Horses. J. Equine Vet. Sci..

[17] Tyler-McGowan C.M., Golland L.C., Evans D.L., Hodgson D.R., Rose R.J. (1999). Haematological and Biochemical Responses to Training and Overtraining. Equine Vet. J. Suppl..

[18] Santos V.P.S., Gonzales F.D.G., Castro Junior F.C., Correio T.F.C. (2015). Hematobiochemical response to exercise with ergometric treadmil, mount training and competition in jumping horses. Arch. Vet. Sci..

[19] Arfuso F., Rizzo M., Giannetto C., Giudice E., Cirincione R., Cassata G., Cicero L., Piccione G. (2022). Oxidant and Antioxidant Parameters’ Assessment Together with Homocysteine and Muscle Enzymes in Racehorses: Evaluation of Positive Effects of Exercise. Antioxidants.

[20] Busechan S., Marchesi M.C., Pieramati C., Forte C., Zappulla F., Conti M.B., Buttarelli D., Rueca F. (2018). Changes in blood parameters in healthy horses and horses with upper and lowe.r respiratory tract diseases undergoing treadmill exercise tests. J. Equine Vet. Sci..

[21] Piccione G., Giannetto C., Fazio F., Mauro S.D., Caola G. (2007). Haematological response to different workload in jumper horses. Bulg. J. Vet. Med..

[22] Piccione G., Giannetto C., Assenza A., Fazio F., Caola G. (2007). Serum electrolyte and protein modification during different workload in jumper horse. Comp. Clin. Pathol..

[23] Fan Y.K., Hsu J.C., Peh H.C., Tsang C.L., Cheng S.P., Chiu S.C., Ju J.C. (2002). The Effects of Endurance Training on the Hemogram of the Horse. Asian-Australas. J. Anim. Sci..

[24] Soroko M., Śpitalniak-Bajerska K., Zaborski D., Poźniak B., Dudek K., Janczarek I. (2019). Exercise-Induced Changes in Skin Temperature and Blood Parameters in Horses. Arch. Anim. Breed..

[25] Deniz Ö., Aragona F., Murphy B.A., Tümer K.Ç., Bozacı S., Fazio F. (2024). Climate Change Impact on Blood Haemogram in the Horse: A Three-Year Preliminary Study. Front. Vet. Sci..

[26] Aragona F., Tabbì M., Gugliandolo E., Giannetto C., D’Angelo F., Fazio F., Interlandi C. (2024). Role of Cannabidiolic Acid or the Combination of Cannabigerol/Cannabidiol in Pain Modulation and Welfare Improvement in Horses with Chronic Osteoarthritis. Front. Vet. Sci..

[27] Cruz N.I.D.L., Merino J.O., López E.A., Monreal A.E., Aguirre G., Rangel J.A., Venegas C. (2017). Effect of Age, Gender, and Season on Hematological Parameters in Quarter Horses. J. Vet. Sci. Med. Diagn..

[28] Deniz Ö., Ekinci G., Onmaz A.C., Derelli F.M., Fazio F., Aragona F., van den Hoven R. (2024). Monitoring of Inflammatory Blood Biomarkers in Foals with Rhodococcus Equi Pneumonia during Antimicrobial Treatment. J. Equine Vet. Sci..

[29] Silva C.J.F.L., Trindade K.L.G., Cruz R.K.S., Manso H.E.C.C.C., Coelho C.S., Filho J.D.R., Nogueira C.E.W., Aragona F., Fazio F., Manso Filho H.C. (2022). Effects of the Ingestion of Ripe Mangoes on the Squamous Gastric Region in the Horse. Animals.

[30] Desgorces F.D., Testa M., Petibois C. (2008). Training-Level Induced Changes in Blood Parameters Response to on-Water Rowing Races. J. Sports Sci. Med..

[31] Piccione G., Casella S., Monteverde V., Giannetto C., Caola G. (2008). Haematological Modifications during Official 1600 and 2000 Meters Trot Races in Standardbred Horses. Acta. Vet..

[32] Piccione G., Casella S., Giannetto C., Monteverde V., Ferrantelli V. (2009). Exercise-Induced Modifications on Haematochemical and Electrophoretic Parameters During 1600 and 2000 Meters Trot Races in Standardbred Horses. J. Appl. Anim. Res..

[33] Piccione G., Vazzana I., Giannetto C., Gianesella M., Ferrantelli V. (2008). Modification of Some Haematological and Haematochemical Parameters in Horse During Long Distance Rides. Res. J. Vet. Sci..

[34] Piccione G., Casella S., Giannetto C., Messina V., Monteverde V., Caola G., Guttadauro S. (2009). Haematological and haematochemical responses to training and compatition in standardbred horses. Comp. Hematol. Int..

[35] Assenza A., Tosto F., Piccione G., Fazio F., Nery J., Valle E., Bergero D. (2012). Lipid Utilization Pathways Induced by Early Training in Standardbred Trotters and Thoroughbreds. J. Equine Vet. Sci..

[36] Strapák P., Holly A., Mlyneková E., Topoľčianky S. (2008). Influence stress on the training process of the horses. J. Cent. Eur. Agricul..

[37] Sribhen C., Sitthichaiyakul A., Kongpiromchean Y., Sribhen K. (2007). Influence of Training Exercise on Clinical Plasma Chemistry Parameters and Cardiac Markers in Race Horses. Agric. Nat. Resour..

[38] Padalino B., Rubino G., Centoducati P., Petazzi F. (2007). Training versus Overtraining: Evaluation of Two Protocols. J. Equine Vet. Sci..

[39] Miglio A., Falcinelli E., Mezzasoma A.M., Cappelli K., Mecoci S., Gesele P., Antognoni M.T. (2021). Effect of first long-term tem training on whole blood count and blood clotting parameters in thoroughbreds. Animals.

[40] Miglio A., Falcinelli E., Cappelli K., Mecocci S., Mezzasoma A.M., Antognoni M.T., Gresele P. (2024). Effect of Regular Training on Platelet Function in Untrained Thoroughbreds. Animals.

[41] Aragona F., Arfuso F., Fazio F., De Caro S., Giudice E., Monteverde V., Piccione G., Giannetto C. (2023). Circadian Variation of Peripheral Blood Cells in Horses Maintained in Different Environmental and Management Conditions. Animals.

[42] Giannetto C., Arfuso F., Fazio F., Giudice E., Pietro S.D., Bruschetta D., Piccione G. (2017). Different Training Schedules Influence Platelet Aggregation in Show Jumping Horses. Pol. J. Vet. Sci..

[43] Arfuso F., Giannetto C., Interlandi C., Giudice E., Bruschetta A., Panzera M.F., Piccione G. (2021). Dynamic Metabolic Response, Clotting Times and Peripheral Indices of Central Fatigue in Horse Competing in a 44 km Endurance Race. J. Equine Vet. Sci..

[44] Arfuso F., Rizzo M., Arrigo F., Faggio C., Giudice E., Piccione G., Giannetto C. (2024). Dynamic Correlation between Platelet Aggregation and Inflammatory-like State in Athlete Horses. Appl. Sci..

[45] Smith J.E. (2003). Effects of strenuous exercise on haemostasis. Br. J. Sports Med..

[46] Assenza A., Tosto F., Casella S., Fazio F., Giannetto C., Piccione G. (2013). Changes in Blood Coagulation Induced by Exercise Training in Young Athletic Horses. Res. Vet. Sci..

[47] Bayly W. (1983). Effects of Exercise on the Hemostatic System of Thoroughbred Horses Displaying Post-Exercise Epistaxis. J. Equine Vet. Sci..

[48] Johnstone I.B., Viel L., Crane S., Whiting T. (1991). Hemostatic Studies in Racing Standardbred Horses with Exercise-Induced Pulmonary Hemorrhage. Hemostatic Parameters at Rest and after Moderate Exercise. Can. J. Vet. Res..

[49] Piccione G., Bazzano M., Giannetto C., Marafioti S., Fazio F. (2014). Training-Induced Changes in Clotting Parameters of Athletic Horses. J. Vet. Sci..

[50] Tikhomirova S.V., Vikulov A.D., Baranov A.A., Osetrov I.A. (2007). Plasma-coagulation hemostasis in physically active subjects during adaptation to physical exercise. Hum. Physiol..

[51] Jackson S.P. (2007). The growing complexity of platelet aggregation. Blood.

[52] Rand M.L., Leung R., Packham M.A. (2003). Platelet function assays. Transfus. Apher. Sci..

[53] Piccione G., Assenza A., Borruso M., Fazio F., Caola G. (2009). Daily Pattern of Some Fatty Acids in the Athletic Horse. J. Anim. Physiol. Anim. Nutr..

[54] Assenza A., Arfuso F., Zanghì E., Fazio F., Bruschetta D., Piccione G. (2016). Lipid and lipoprotein profiles modification in athletic horses following repeated jumping events. J. Equine Vet. Sci..

[55] Westermann C.M., Dorland B., de Sain-van der Velden M.G., Wijnberg I.D., Van Breda E., De Graaf-Roelfsema E., Keizer H.A., Van der Kolk J.H. (2008). Plasma Acylcarnitine and Fatty Acid Profiles during Exercise and Training in Standardbreds. Am. J. Vet. Res..

[56] Piccione G., Giannetto C., Costa C., Fazio F., Caola G. (2007). Effects of high intensity exercise on serum electrolytes and protein in Thoroughbred horses. Magy. Allatorvosok Lapja.

[57] Kaneko J.J., Harwey J.W., Bruss M.L. (2008). Clinical Biochemistry of Domestic Animals.

[58] Fazio F., Assenza A., Tosto F., Casella S., Piccione G., Caola G. (2011). Training and haematochemical profile in Thoroughbreds and Standardbreds: A longitudinal study. Livestock Sci..

[59] Piccione G., Assenza A., Casella S., Giannetto C., Tosto F., Caola G. (2010). Modification of platelet aggregation during treadmill section and obstacle course in athletic horses. Acta. Vet..

[60] Fontenot R.L., Sink A., Were S.R., Weinstein N.M., Dahlgren L.A. (2012). Site tube centrifugation for processing platelet-rich plasma in the horse. Can. Vet. J..

[61] Hodgson D.R., Rose R.J. (1994). The athletic horse. Principles and Practice of Equine Sports Medicine.

[62] Sawka M.N., Convertino V.A., Eicher E.R., Schneider S.M., Young A.J. (2000). Blood olume: Importance and adatations to exercise training, environmental stress and trauma/sickness. Med. ScinSports Exerc..

[63] Cebulj-Kadunc N., Bozic M., Kosec M., Cestnik V. (2002). The influence of age and gender on haematological parameters in Lipizzan horses. J. Vet. Med. A Physiol. Pathol. Clin. Med..

[64] Linder A., Signorini R., Brero L., Arn E., Mancini R., Enrique A. (2006). Effect of conditioning horses with short intervals at high speed on biochemical variables in blood. Equine Vet. J..

[65] Couroucè A. (1991). Field exercise testing for assessing fitness in French standardbred trotters. Vet. J..

[66] Padalino B., Frate A., Tateo A., Siniscalchi M., Quaranta A. (2005). Valutazione dello stato di preparazione atletica del cavallo trottatore su pista dritta mediante determinazione del lattato, del valore ematocrito e di alcuni parametri fisiologici. Ippologia.

[67] Assenza A., Gongiu F., Giannetto C., Fazio F., Piccione G. (2015). Haematological response associated with repeated show jumping competition in horses. Acta. Sci. Vet..

[68] Fedde M.R., Wood S.C. (1993). Rheological characteristics of horse blood: Significance during exercise. Resp. Physiol..

[69] Kjeldsen S.E., Weder A.B., Egan V., Neubig R., Zweifler A.J., Julòius S. (1995). Effect of circulating epinephrine on platelet function and haematocrit. Hypertension.

[70] Nesbitt W.S., Westein E., Tovar-Lopez F.J., Tolouei E., Mitchell A., Fu J., Carberry J., Fouras A., Jackson S.P. (2009). A shear gradient-dependent platelet aggregation mechanism drives thrombus formation. Nat. Med..

[71] Arfuso F., Giannetto C., Giudice E., Fazio F., Piccione G. (2016). Dynamic Modulation of Platelet Aggregation, Albumin and Nonesterified Fatty Acids during Physical Exercise in Thoroughbred Horses. Res. Vet. Sci..

[72] Pösö A.R., Viljanen-Tarifa E., Soveri T., Oksanen H.E. (1989). Exercise-Induced Transient Hyperlipidemia in the Racehorse. Zentralbl. Veterinarmed. A.

[73] Dhindsa S., Ghanim H., Dandona P. (2015). Nonesterified Fatty Acids, Albumin, and Platelet Aggregation. Diabetes.

[74] Bartolomé E., Perdomo-González D.I., Sánchez-Guerrero M.J., Valera M. (2021). Genetic Parameters of Effort and Recovery in Sport Horses Assessed with Infrared Thermography. Animals.

[75] Adamu L., Noraniza M.A., Rasedee A., Bashir A. (2013). Effect of Age and Performance on Physical, Hematological, and Biochemical Parameters in Endurance Horses. J. Equine Vet. Sci..

